# Hepatogenous diabetes in the era of precision medicine: diagnosis, management, and future directions

**DOI:** 10.1007/s10238-026-02112-8

**Published:** 2026-04-19

**Authors:** Gasser El-Azab, Mohamed Akl Rady, Medhat Assem, Hanaa Nagdy

**Affiliations:** 1 Present Address: Hepatology and Gastroenterology Department, National Liver Institute - Menoufia University, Shebeen El-Kom, Egypt; 2 Internal Medicine Department, Faculty of Medicine, University of Bisha, Bisha, Saudi Arabia; 3 Internal Medicine Department, College of Medicine, Arab academy for science and technology and maritime transport, Alexandria, Egypt

**Keywords:** Hepatogenous diabetes, Cirrhosis, Glucose intolerance, Insulin resistance, β-cell dysfunction, Gut microbiota, Hepatokines, Sarcopenia, Steatohepatitis

## Abstract

Hepatogenous diabetes (HD) is a distinct clinical entity that arises as a direct consequence of chronic liver disease, particularly cirrhosis. The complex interplay between hepatic dysfunction and glucose metabolism gives rise to unique pathophysiological mechanisms, including severe hepatic insulin resistance, pancreatic β-cell dysfunction, chronic inflammation, oxidative stress, and alterations in gut microbiota. HD is frequently underdiagnosed due to the limitations of traditional diagnostic criteria, as patients often exhibit normal fasting glucose and HbA1c levels despite significant postprandial hyperglycemia. The oral glucose tolerance test is more sensitive for detecting HD in this population. HD differs from type 2 diabetes mellitus in its temporal relationship with liver disease, absence of classic metabolic risk factors, greater glycemic variability, and a lower prevalence of microvascular complications. The prevalence of HD increases with the severity of liver dysfunction and varies according to the underlying etiology, with the highest rates observed in metabolic-associated fatty liver disease and hemochromatosis. HD is associated with accelerated liver fibrosis, increased risk of hepatocellular carcinoma, higher rates of hepatic decompensation, and increased mortality. Management strategies require careful consideration of the altered pharmacokinetics and increased risk of hypoglycemia in advanced liver disease. Individualized management strategies, including risk stratification and targeted therapies, hold promise for improving outcomes. This review synthesizes current knowledge on the epidemiology, pathophysiology, clinical implications, diagnosis, and management of HD, and highlights areas for future research to enhance recognition and treatment of this important but often overlooked complication of chronic liver disease.

## Introduction

Diabetes mellitus (DM) and chronic liver disease (CLD) represent two major global health challenges with significant morbidity and mortality. The liver plays a crucial role in glucose homeostasis through the regulation of multiple metabolic pathways that maintain blood glucose levels within a narrow physiological range [1]. Impairment of liver function profoundly disrupts these regulatory mechanisms. The diabetogenic potential of liver cirrhosis has been recognized for over a century, with the term “hepatogenous diabetes” (HD) first coined by Naunyn in 1906 to describe diabetes that develops as a consequence of liver cirrhosis [2–4].

The relationship between liver disease and diabetes is bidirectional. While liver cirrhosis can lead to the development of HD, pre-existing diabetes can contribute to the development and progression of liver diseases such as metabolic dysfunction–associated steatotic liver disease (MASLD) [5]. This complex interrelationship creates diagnostic challenges, particularly in early cirrhosis, as both conditions have indolent courses, making it difficult to determine which condition appeared first [6].

The lack of universally accepted diagnostic criteria has hindered both clinical recognition and research comparability. In this review, we summarize and integrate current evidence on HD, addressing its definition, pathophysiological mechanisms, clinical implications, diagnostic approaches, and management strategies across the spectrum of liver disease. We also discuss existing controversies, highlight knowledge gaps, and outline future research priorities to improve the understanding and management of this underrecognized condition.

## Relationship between liver and diabetes

### Normal glucose homeostasis and the role of the liver

The liver is central to glucose homeostasis through coordinated regulation of hepatic glucose production, uptake, storage, and release [7]. During fasting, euglycemia is maintained by glycogenolysis and gluconeogenesis, whereas in the postprandial state the liver takes up glucose from the portal circulation, inhibits glucose production, and stores glycogen [8]. These processes are primarily regulated by insulin and glucagon, with insulin inhibiting hepatic glucose output and stimulating glycogenesis, while glucagon promotes glycogenolysis and gluconeogenesis [9].

The liver is also the major site of insulin clearance, removing approximately 50–80% of portal insulin during first-pass metabolism [10]. This mechanism is crucial for regulating systemic insulin levels and peripheral insulin action. In addition, the liver secretes several hepatokines, including insulin-like growth factor-1 (IGF-1), fibroblast growth factor 21 (FGF21), and fetuin-A, which modulate whole-body glucose metabolism and insulin sensitivity [11, 12].

## Pathophysiological mechanisms linking liver disease and diabetes

### Insulin resistance

Insulin resistance (IR) is a central feature of both type 2 diabetes mellitus (T2DM) and HD, but is typically more severe in the latter [4]. In cirrhosis, IR arises from impaired hepatic insulin clearance due to hepatocellular dysfunction and portosystemic shunting, resulting in peripheral hyperinsulinemia and downstream receptor and post-receptor signaling defects [13]. Persistent systemic inflammation, oxidative stress, and endotoxemia related to portal hypertension and increased intestinal permeability further disrupt insulin signaling through cytokine activation, stress-sensitive kinases, impaired GLUT4 translocation, and mitochondrial dysfunction [14–16]. In parallel, dysregulated hepatokine secretion, characterized by increased fetuin-A and reduced FGF21, exacerbates insulin resistance in advanced liver disease [11, 12, 17].

### Pancreatic β-cell dysfunction

Progression from impaired glucose tolerance to overt hepatogenous diabetes requires pancreatic β-cell dysfunction. Impaired hepatic clearance of circulating toxins, chronic glucotoxicity, and portal hypertension–related pancreatic congestion and hypoxia contribute to progressive β-cell injury and reduced insulin secretion [18, 19]. In selected etiologies, particularly hepatitis C virus infection, direct viral involvement of pancreatic tissue may further impair β-cell function [20]. Overt diabetes develops when β-cell compensation is no longer sufficient to counterbalance severe hepatic insulin resistance [21].

### Gut dysbiosis

Cirrhosis is associated with significant dysbiosis characterized by reduced microbial diversity and overgrowth of potentially pathogenic bacteria [22]. This contributes to increased intestinal permeability, altered bile acid metabolism, and reduced production of beneficial short-chain fatty acids, all of which affect glucose metabolism [23, 24].

### Hyperammonemia

Elevated ammonia levels in cirrhosis interfere with insulin signaling, impair glucose transport, and contribute to mitochondrial dysfunction [25].

### Sarcopenia and myosteatosis

Loss of skeletal muscle mass and increased intramuscular fat deposition (Myosteatosis) are common in cirrhosis and contribute significantly to IR and glucose dysregulation [26, 27].

### Hepatokine and adipokine dysregulation

Altered production of liver-derived proteins (hepatokines) and adipose tissue-derived hormones (adipokines) creates a unique metabolic environment in cirrhosis that contributes to HD development [28, 29].

**Fig. 1:**
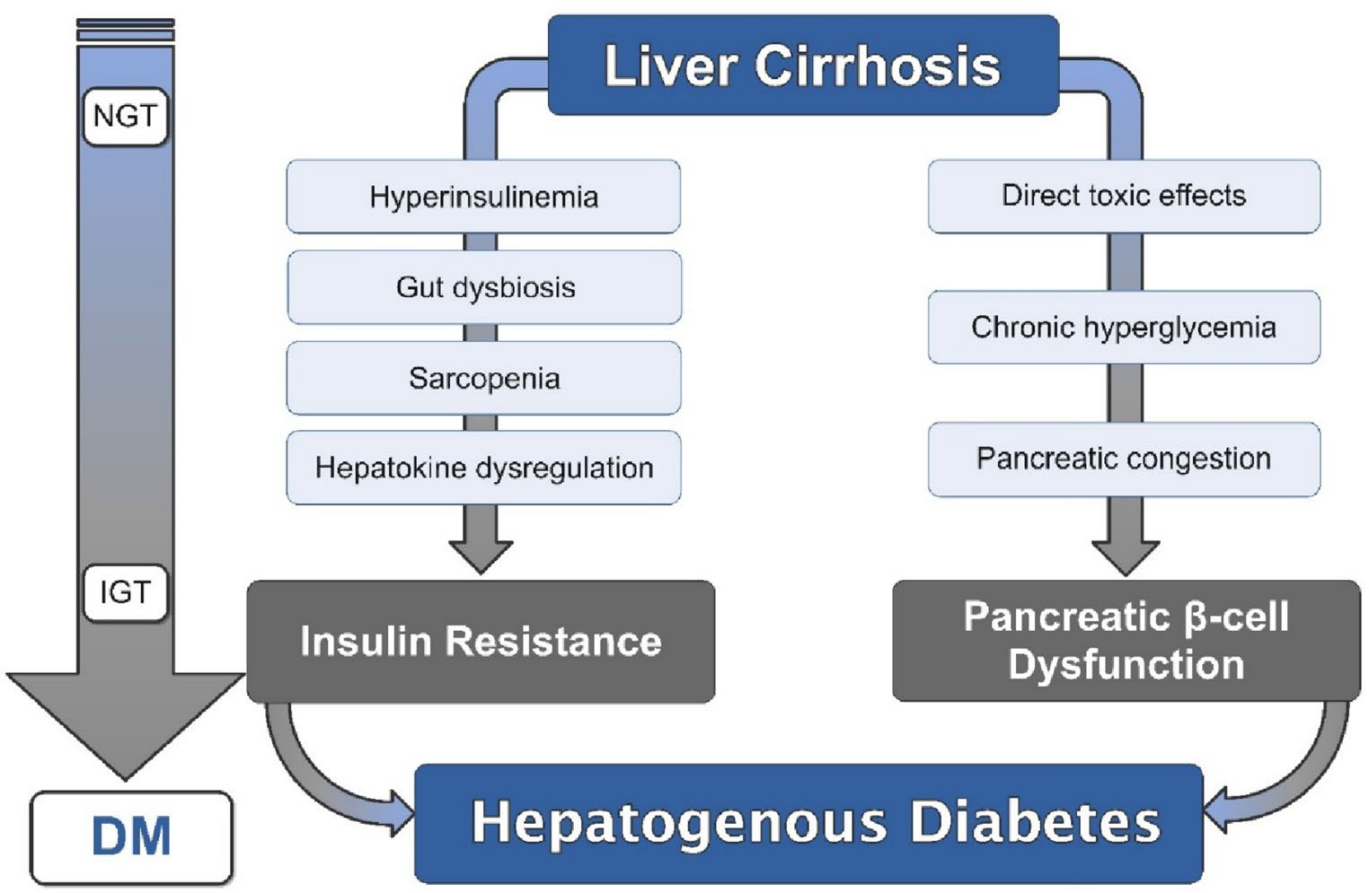
Pathophysiological mechanisms underlying the development of hepatogenous diabetes in patients with liver cirrhosis. This schematic illustrates how chronic liver injury and cirrhosis promote hepatogenous diabetes (HD) through two interrelated pathways. (1) Hepatic insulin resistance arises from portal–systemic shunting, hyperinsulinemia, chronic inflammation, gut dysbiosis, sarcopenia, and altered hepatokine secretion (e.g., decreased IGF-1, increased fetuin-A and FGF21). (2) Pancreatic β-cell dysfunction develops secondary to toxic metabolic stress, oxidative injury, chronic hyperglycemia, and pancreatic venous congestion. The combined effects of impaired insulin action and secretion lead to progressive disturbances in glucose metabolism, evolving from normal glucose tolerance (NGT) to impaired glucose tolerance (IGT) and ultimately to overt diabetes mellitus (DM)

Figure 1 illustrates the key pathophysiological pathways of hepatogenous diabetes in liver cirrhosis.

## Bidirectional relationship between liver disease and diabetes

The relationship between liver disease and diabetes is bidirectional, creating complex interactions and potential vicious cycles [30, 31]:

### Effects of Liver Disease on Diabetes Risk and Control

1. **Progression from impaired glucose tolerance (IGT) to diabetes**: As liver function deteriorates, the risk of developing diabetes increases. Studies have shown that the incidence of diabetes is significantly higher in patients with worsening Child-Pugh class during follow-up [2].
2. **Etiology-specific diabetogenic effects**: Diabetes risk varies by liver disease etiology and is particularly high in MASLD–related, cryptogenic, hepatitis C virus–related, and alcoholic cirrhosis [32]. In HCV infection, viral proteins directly impair insulin signaling, while viral eradication with direct-acting antivirals is associated with improved glucose metabolism [33].
3. **Glycemic variability**: Liver disease impairs glycogen storage and gluconeogenesis, leading to increased glycemic variability and risk of hypoglycemia, particularly in advanced cirrhosis [34].
4. **Vascular complications**: The presence of liver disease, especially MASLD or metabolic dysfunction–associated steatohepatitis (MASH), significantly exacerbates the risk and progression of both microvascular and macrovascular complications in diabetes [35].

### Effects of Diabetes on Liver Disease Progression and Outcomes

1. **Accelerated fibrosis progression**: Diabetes promotes liver fibrosis through multiple mechanisms including increased oxidative stress, lipotoxicity, and activation of hepatic stellate cells [36–39].
2. **Increased risk of hepatocellular carcinoma (HCC)**: Diabetes is an independent risk factor for HCC development, with hyperinsulinemia promoting hepatocyte proliferation and inhibiting apoptosis [40, 41].
3. **Higher rates of decompensation**: Diabetic patients with cirrhosis have higher rates of hepatic decompensation, including ascites, variceal bleeding, and hepatic encephalopathy (HE) [42, 43].
4. **Increased mortality**: Diabetes significantly increases mortality in patients with cirrhosis, with a hazard ratio of 1.5-2.0 compared to non-diabetic cirrhotic patients [44, 45].
5. **Reduced response to antiviral therapy**: In HCV infection, diabetes has been associated with reduced sustained virological response rates to interferon-based therapies [46].
6. **Post-transplant complications**: Pre-existing diabetes and post-transplant diabetes mellitus (PTDM) are associated with increased risk of infection, cardiovascular events, and graft failure after liver transplantation [47].

## Hepatogenous diabetes

### Definition

HD is defined as diabetes mellitus that develops as a direct consequence of liver cirrhosis or severe liver dysfunction [48]. For HD to be diagnosed, diabetes must have occurred after the onset of cirrhosis, distinguishing it from pre-existing T2DM [3].

### Diagnostic criteria

Currently, there are no universally accepted diagnostic criteria specifically for HD, and the condition is often diagnosed using standard criteria for diabetes mellitus. However, these may underestimate HD, as patients with cirrhosis frequently have normal fasting glucose and HbA1c despite significant postprandial hyperglycemia [49].

Clinically, HD should be suspected in patients with established cirrhosis who lack classical metabolic risk factors for T2DM and who demonstrate normal fasting glucose and/or HbA1c in the presence of postprandial hyperglycemia. In this setting, the oral glucose tolerance test (OGTT) is the most sensitive diagnostic tool, as it detects abnormalities that routine measurements may miss [31, 50].

### Proposed diagnostic framework for hepatogenous diabetes

Given the difficulty in establishing the temporal relationship between diabetes and cirrhosis, and the limitations of standard glycemic markers, we propose the following pragmatic diagnostic framework:

1. **Confirmed cirrhosis**: Diagnosis established by clinical, imaging, elastographic, or histological criteria.
2. **Temporal relationship**: Onset of diabetes occurring after the diagnosis of cirrhosis. In newly diagnosed cirrhosis, baseline diabetes screening is essential to document this relationship.
3. **Limited metabolic phenotype**: Absence or minimal features of metabolic syndrome or strong family history of T2DM.
4. **Postprandial glycemic abnormality**: OGTT is preferred; a 2-hour plasma glucose ≥ 200 mg/dL following a 75 g glucose load confirms diabetes.
5. **Association with liver disease severity**: Glycemic abnormalities that parallel worsening hepatic dysfunction support the diagnosis of HD.

It should be emphasized that in stable outpatients with compensated cirrhosis and preserved synthetic liver function, the application of standard American Diabetes Association (ADA) diagnostic criteria for diabetes mellitus: (FBS ≥ 126 mg/dL, or HbA1c ≥ 6.5%) remains reasonable and clinically appropriate, provided their limitations are recognized [51].

### Differences between hepatogenous diabetes and type 2 diabetes mellitus

HD and T2DM share features of dysglycemia but differ markedly in pathogenesis, clinical profile, prognosis, and management [50]. Distinguishing HD from T2DM has important clinical implications, as HD reflects liver disease severity, predicts hepatic decompensation and mortality, and often improves after liver transplantation, unlike T2DM [52, 53]. Recognition of HD enables individualized glycemic targets, safer drug selection, and focused monitoring of liver-related outcomes. Key differences between HD and T2DM are summarized in Table 1.

**Table 1:** Distinguishing Features of Hepatogenous Diabetes and Type 2 Diabetes Mellitus in Patients with Liver Disease [53–56]

Characteristic	Hepatogenous Diabetes	Type 2 Diabetes Mellitus in liver cirrhosis
Definition	Diabetes that develops as a consequence of chronic liver disease, especially cirrhosis	Pre-existing T2DM that coincides with or contributes to liver disease
Temporal relationship	Develops *after* onset of cirrhosis	Often *precedes* liver disease
Prevalence	Affects approximately 20–60% of patients with cirrhosis, depending on disease etiology and severity.	Present in 20–30% of cirrhotic patients
Metabolic Risk Factors	Typically absent; not strongly associated with metabolic risk factors.	Frequently associated with metabolic syndrome (obesity, dyslipidemia, family history)
Insulin Resistance	Marked insulin resistance due to liver dysfunction	Insulin resistance is present but generally less severe
Clinical Features	Often asymptomatic or mild symptoms, may be masked by or attributed to underlying liver disease	Classic diabetic symptoms (polyuria, polydipsia, polyphagia)
Glycemic Pattern	Predominant postprandial hyperglycemia; normal fasting glucose common.	Fasting and postprandial hyperglycemia; typically more severe and persistent.
Hypoglycemia Risk	Higher risk, especially during fasting or with certain medications	Less prone to hypoglycemia
Severity Correlation	Prevalence and severity correlate strongly with Child-Pugh class/MELD score	Not directly related to liver function
Vascular Complications	Lower incidence of microvascular and macrovascular complications	Higher risk of complications due to longer disease duration and metabolic comorbidities
Post-Liver Transplant	Often improves or resolves after liver transplantation.	Typically persists and may worsen post-transplant due to immunosuppressive therapy

### HD and MASLD-Related T2DM Overlap

Distinguishing HD from T2DM becomes particularly challenging in cirrhosis related to MASLD. MASLD is fundamentally driven by insulin resistance, a core feature of T2DM. In this context, T2DM and MASLD-related cirrhosis often co-exist, making it difficult to determine the primary driver of glucose intolerance [57].

MASLD-related cirrhosis often represents a mixed phenotype in which classical metabolic insulin resistance coexists with cirrhosis-induced impairments in hepatic glucose handling. In such patients, the dominant driver of dysglycemia may shift over time from metabolic syndrome–related mechanisms to liver failure–related mechanisms. Recognizing this continuum has important implications for prognosis and therapeutic decision-making, as hepatogenous features are more closely linked to hepatic decompensation and may improve following liver transplantation.

### Prevalence of hepatogenous diabetes

The prevalence of HD varies widely according to liver disease etiology, diagnostic criteria, and severity of cirrhosis [58]. Overall, HD is substantially more common in patients with cirrhosis than diabetes in the general population, where prevalence is estimated at approximately 10.5% [59].

In studies specifically examining HD prevalence using OGTT, rates range from 21% to 57% [36, 60, 61]. When diagnosed based on clinical history alone (onset of diabetes after diagnosis of cirrhosis), lower prevalence rates of 15.9% to 29.2% have been reported [62, 63], highlighting the importance of OGTT in detecting HD.

### Relationship with severity of liver disease

HD is closely linked to the severity of liver dysfunction. As liver disease progresses, the prevalence and severity of HD increase, reflecting a stronger diabetogenic potential in advanced cirrhosis. In a prospective study, patients with compensated cirrhosis and normal baseline glucose tolerance developed diabetes in 4.4% and 21.2% of cases after one and four years, respectively, with higher rates observed among those whose Child-Pugh class worsened during follow-up [64]. Cross-sectional data further show that diabetes prevalence rises markedly across Child-Pugh classes A (20.5%), B (56%), and C (61%) [65].

HD is also significantly associated with higher MELD scores (> 15), presence of large varices, and hepatocellular carcinoma [61]. Similarly, elevated hepatic venous pressure gradient (HVPG) has been shown to increase the risk of HD (OR = 1.15) [42]. The presence of portal hypertension complications, such as esophageal varices and ascites, further correlates with impaired glucose metabolism [42]. The worsening diabetogenic potential of cirrhosis in parallel with disease severity indicates a detrimental impact of liver failure on glucose metabolism that extends beyond the simple association of the two conditions [3].

## Management of hepatogenous diabetes

### General principles

The management of HD presents unique challenges due to the complex interplay between impaired liver function and disrupted glucose metabolism. Currently, there are no specific clinical guidelines tailored to the management of HD. As a result, treatment is generally adapted from T2DM guidelines, with necessary adjustments based on the severity of liver disease. This requires close collaboration between hepatologists, endocrinologists, and dietitians to optimize patient outcomes while minimizing the risk of adverse effects [66].

### Goals of therapy

The primary goals of HD management are to achieve and maintain appropriate glycemic control while minimizing the risk of hypoglycemia; prevent both diabetes-related complications and liver-related complications; preserve liver function by avoiding hepatotoxic medications; individualize treatment based on the severity of liver disease, nutritional status, comorbidities, and patient preferences; and address the underlying pathophysiological mechanisms of HD.

Glycemic targets should be individualized based on the patient’s clinical status, risk of hypoglycemia, and life expectancy. In general, less stringent glycemic targets (HbA1c 7.5-8.0%) may be appropriate for patients with advanced liver disease, multiple comorbidities, or limited life expectancy, while more stringent targets (HbA1c < 7.0%) may be suitable for patients with compensated cirrhosis and longer life expectancy [67].

### Lifestyle modifications and nutritional considerations

Lifestyle modifications are a cornerstone of diabetes management and play a critical role in patients with HD. However, they require specialized adjustments in the context of liver disease due to altered metabolic demands, the risk of malnutrition, and potential complications such as HE [68].

#### Dietary recommendations

Dietary management in cirrhotic patients with diabetes differs significantly from that of the general T2DM population. Caloric restriction is generally not recommended in cirrhosis due to the high prevalence of malnutrition and sarcopenia. Most patients require an energy intake of 25–35 kcal/kg/day to maintain metabolic stability and prevent catabolic states. Protein intake should be maintained at 1.2–1.5 g/kg/day to preserve lean body mass and prevent sarcopenia, except during acute episodes of HE, when temporary protein restriction may be warranted. To reduce glycemic fluctuations, carbohydrate intake should be evenly distributed across multiple small meals throughout the day. A late evening snack containing complex carbohydrates and protein is advised to minimize nocturnal hypoglycemia and reduce overnight protein catabolism. Importantly, complete abstinence from alcohol is essential for all patients, regardless of the underlying etiology of liver disease [68].

#### Physical activity

Physical activity offers several benefits for patients with HD, including improved insulin sensitivity, reduction of portal pressure, and preservation of muscle mass. A regimen of low- to moderate-intensity exercise, such as walking, swimming, or cycling, for 30 min on most days of the week (3–5 times) is generally safe and effective [69]. However, patients with severe portal hypertension, recent variceal bleeding, or tense ascites should avoid high-intensity exercise and refrain from heavy lifting due to the risk of complications.

#### Weight management

Weight management must be carefully tailored to the patient’s nutritional and hepatic status. In obese patients with compensated cirrhosis, particularly those with MASLD or MASH, gradual weight loss (0.5–1 kg/week) may improve both insulin sensitivity and liver function [70]. Conversely, in malnourished individuals, efforts should focus on nutritional supplementation and anabolic strategies to restore muscle mass and prevent further decline. Special attention is required for patients with sarcopenic obesity, a condition characterized by concurrent obesity and muscle loss. These patients benefit from a balanced approach that targets fat loss while preserving or enhancing lean muscle mass [71].

### Pharmacological therapy

The selection of antidiabetic agents in HD is complex, requiring a careful balance between glycemic efficacy and clinical safety, particularly concerning the risk of hypoglycemia and drug accumulation due to impaired hepatic metabolism. The benefits, limitations, and specific recommendations for antidiabetic drugs in patients with liver disease are summarized in Table 2.

#### Insulin and insulin analogs

Insulin remains the cornerstone of diabetes management in advanced liver disease. Because human insulin is primarily metabolized by the liver, its clearance may be reduced in cirrhosis, potentially leading to prolonged action and an increased risk of hypoglycemia. Insulin analogs are generally preferred as they offer more predictable pharmacokinetics and are less affected by fluctuating liver function [72].

Insulin requirements can be high in compensated cirrhosis due to severe insulin resistance. However, as liver disease progresses to decompensation, requirements often decrease due to reduced hepatic gluconeogenesis and improved peripheral insulin sensitivity [73].

A basal-bolus insulin regimen is preferred for most hospitalized patients and those with advanced liver disease, as it provides greater flexibility and tighter glycemic control. Premixed insulin formulations are generally discouraged due to limited dosing flexibility and a higher risk of hypoglycemia. Insulin therapy has been linked to an increased risk of HCC and should be avoided in patients with high-risk features, such as dysplastic nodules and elevated alpha-fetoprotein levels [74].

#### Oral antidiabetic agents

The use of oral agents is highly dependent on the Child-Pugh class and the presence of cirrhosis-related complications.

- **Metformin**: Holds a unique position due to its potential hepatoprotective effects, including a reduced risk of hepatocellular carcinoma and improved insulin sensitivity [75]. It is safely used in compensated cirrhosis (Child-Pugh A) and early Child-Pugh B if renal function is preserved, but must be avoided in decompensated cirrhosis due to the risk of lactic acidosis [76].
- **Sulfonylureas and Glinides**: These agents are generally avoided in cirrhosis due to extensive hepatic metabolism and a significantly increased risk of severe, prolonged hypoglycemia [77]. If absolutely necessary, short-acting agents at reduced doses may be considered in Child-Pugh A cirrhosis with close monitoring [78].
- **Thiazolidinediones**: Pioglitazone may benefit MASLD-related cirrhosis by improving insulin sensitivity and potentially reducing fibrosis [79, 80]. However, the risk of fluid retention may exacerbate ascites and increase heart failure risk [81]. Therefore, it may be considered only in carefully selected patients with compensated Child-Pugh A MASLD-cirrhosis.
- **Dipeptidyl Peptidase-4 (DPP-4) Inhibitors**: These agents enhance glucose-dependent insulin secretion without typically causing hypoglycemia or weight gain [82]. They are generally considered safe in mild-to-moderate liver dysfunction (Child-Pugh A–B), with linagliptin being preferred due to its minimal renal excretion. Caution is required due to reported hepatotoxicity, particularly with vildagliptin, and increased risks of hepatic decompensation [83].
- **Glucagon-like Peptide-1 (GLP-1) Receptor Agonists**: These agents improve hyperglycemia, reduce insulin resistance, and promote weight loss, making them attractive for MASLD-cirrhosis [84, 85]. Notably, semaglutide has received accelerated FDA approval (August 2025) for non-cirrhotic MASH with advanced fibrosis. They may be used in Child-Pugh A and B cirrhosis, though caution is required in patients with severe malnutrition or sarcopenia due to their appetite-suppressant effects [86].
- **Sodium-Glucose Cotransporter 2 (SGLT2) Inhibitors**: These offer cardioprotective and renoprotective benefits and may alleviate fluid retention through mild osmotic diuresis [87, 88]. They are generally considered in Child-Pugh A and early B cirrhosis but should be avoided in advanced disease (Child-Pugh C) due to the risk of volume depletion and acute kidney injury [89, 90].
- **Alpha-Glucosidase Inhibitors (AGIs)**: Acarbose is minimally absorbed and not hepatically metabolized, making it safe across all cirrhosis stages for postprandial hyperglycemia [91]. It may also improve mild hepatic encephalopathy [92]. Efficacy is modest with a low hypoglycemia risk, but gastrointestinal side effects may limit long-term tolerability [93].

**Table 2:** Benefits and Limitations of Antidiabetic Agents in Liver Disease

Drug Class	Pros	Cons	Recommendations
Insulin	- Most effective glucose control - Safe in advanced disease	- High hypoglycemia risk - Weight gain - Potential link to HCC risk - May increase cardiovascular events	- Safest option in advanced cirrhosis (Child-Pugh B/C) - Use analogs for predictable action
Metformin	- Cardioprotective - Reduces HE and HCC risk - Improves insulin resistance - Increases survival	- Rare lactic acidosis risk (esp. if renal impairment) - GI side effects	- First-line in Child-Pugh A/early B with eGFR ≥ 30 mL/min - Avoid in decompensated cirrhosis, renal impairment
Sulfonylureas (e.g., Glipizide, Glyburide, Glimepiride, Gliclazide)	- Potent glucose-lowering	- High hypoglycemia risk - Hepatic metabolism - increase HCC risk - Weight gain	- Generally avoid in all stages of cirrhosis - If absolutely necessary, use short-acting agents (e.g., glipizide) at very low doses, restricted to Child-Pugh A cirrhosis, with close monitoring
Meglitinides (e.g., repaglinide, nateglinide)	- Short-acting - Lower risk of prolonged hypoglycemia - Target postprandial hyperglycemia.	- Hepatic metabolism; - Drug accumulation and hypoglycemia risk in cirrhosis.	- Generally avoid - If essential, short-acting agents (e.g., repaglinide) at reduced doses may be considered in Child-Pugh A cirrhosis with strict monitoring
TZDs (e.g., pioglitazone)	- Improves MASH histology - Low hypoglycemia risk - Reduce HCC risk	- Fluid retention/edema - Weight gain - Long-term safety concerns	- Consider in compensated MASH-cirrhosis (Child-Pugh A) *without* heart failure, obesity, or edema - Avoid in decompensated cirrhosis
DPP-4 Inhibitors (e.g., linagliptin, sitagliptin, saxagliptin, vildagliptin)	- Weight-neutral - Low hypoglycemia risk - May reduce hepatic inflammation	- Hepatic metabolism (except linagliptin) - possible link to decompensation/ variceal bleed risk - Limited advanced cirrhosis data	- Use with dose adjustments in Child-Pugh A/B. Linagliptin preferred (no dose adjustment) - Monitor for decompensation
GLP-1 RAs (e.g., liraglutide, semaglutide, dulaglutide)	- Weight loss - Improves MASH fibrosis/steatosis - Low hypoglycemia risk - Non-hepatic clearance	- Nausea/appetite loss - Limited data in advanced cirrhosis - Cost	- Use in Child-Pugh A/B - beneficial for MASLD - Avoid in history of pancreatitis, gastroparesis, or severe malnutrition/sarcopenia
SGLT2 Inhibitors (e.g.,empagliflozin, dapagliflozin, canagliflozin)	- Weight loss - Cardio/renoprotective - Improves MASH biomarkers - Low hypoglycemia risk	- Increase Genital/UTI risk - Volume depletion risk (AKI/HRS) - Limited advanced cirrhosis data	- Use in Child-Pugh A/early B *with* preserved renal function - Monitor volume status closely - Avoid in recurrent UTIs, ketoacidosis history, or concomitant diuretics
AGIs (e.g., Acarbose)	- No hepatic metabolism - Low hypoglycemia risk - May improve HE	- Modest efficacy - GI side effects (flatulence, diarrhea)	- Safe across all cirrhosis stages (Child-Pugh A–C) for postprandial hyperglycemia

AGIs: Alpha-Glucosidase Inhibitors; DPP-4 inhibitors: dipeptidyl peptidase-4 inhibitors; GLP-1 RAs: glucagon-like peptide-1 receptor agonists; HCC: hepatocellular carcinoma; HE: hepatic encephalopathy; MASH: metabolic dysfunction–associated steatohepatitis; MASLD: Metabolic Dysfunction–Associated Steatotic Liver Disease; SGLT2 inhibitors: sodium-glucose cotransporter 2 inhibitors; TZDs: Thiazolidinediones; UTI: urinary tract infection.

### Precision-based selection of antidiabetic agents in patients with liver cirrhosis

There is a pressing need for precision in selecting antidiabetic therapy for patients with cirrhosis, as treatment decisions must consider multiple clinical factors beyond liver dysfunction alone. The guiding principle is to provide individualized therapy that achieves optimal glycemic control while minimizing adverse effects and preventing hepatic decompensation.

#### Key factors influencing antidiabetic drug selection in cirrhosis

##### Stage of Cirrhosis

The severity of liver disease critically impacts drug safety. In compensated cirrhosis (Child-Pugh A), most antidiabetic agents can be considered. In contrast, decompensated cirrhosis (Child-Pugh B/C) significantly limits options due to heightened risks of hypoglycemia, impaired metabolism, and lactic acidosis, requiring cautious or contraindicated use of certain drugs.

##### Etiology of Cirrhosis

The underlying cause of cirrhosis also influences therapeutic choices. In MASLD- or MASH-related cirrhosis, where insulin resistance plays a central role, insulin-sensitizing agents such as metformin and pioglitazone may provide additional benefits.

##### Presence of Complications

Common cirrhosis complications guide therapy selection. Sulfonylureas and glinides should be avoided in hepatic encephalopathy due to hypoglycemia and neurocognitive risk. Pioglitazone is contraindicated in patients with ascites or fluid overload. Renal impairment (eGFR < 30) necessitates dose adjustments or avoidance of metformin, GLP-1 receptor agonists, and SGLT2 inhibitors. In sarcopenia or malnutrition, drugs that promote weight loss or worsen muscle catabolism should be used cautiously or avoided.

##### Risk of Hepatocellular Carcinoma

Certain agents may influence HCC risk. Metformin is associated with reduced HCC incidence, while sulfonylureas and insulin may promote carcinogenesis and should be used cautiously in high-risk patients [74, 94].

#### Pharmacological Management Stratified by Liver Disease Severity

**Fig. 2:**
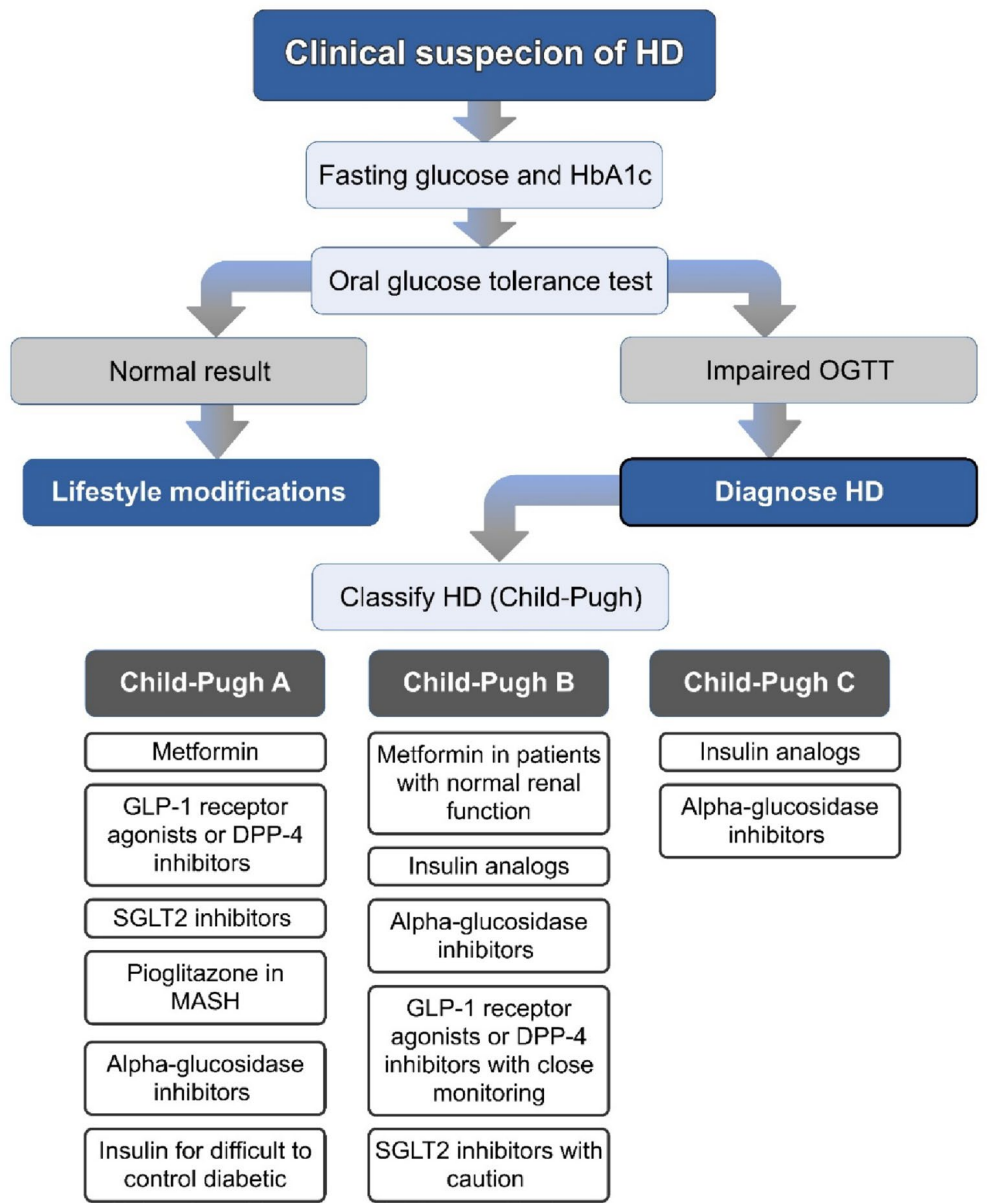
Algorithm for diagnosis and management of hepatogenous diabetes. HD: Hepatogenous Diabetes, OGTT: oral glucose tolerance test

The choice of pharmacological therapy for HD should be guided by the severity of liver disease [95]. A proposed algorithm (Fig. 2) summarizes the stepwise approach to the diagnosis and management of HD.

##### Patients with mild liver disease (Child-Pugh A)

Patients with mild liver disease and preserved synthetic function have the widest range of therapeutic options: Metformin remains the first-line agent. If additional glycemic control is required, GLP-1 receptor agonists or DPP-4 inhibitors may be used safely with appropriate dose adjustments. Vildagliptin is generally avoided, while linagliptin is preferred due to its minimal renal clearance. SGLT2 inhibitors may be beneficial, particularly in patients with volume overload. Pioglitazone may be considered in patients with MASH. Insulin therapy is reserved for patients who fail to achieve glycemic targets or have contraindications to other medications.

##### Patients with moderate liver disease (Child-Pugh B)

As hepatic function declines, the safety and metabolism of many antidiabetic agents become less predictable. Insulin therapy, preferably using analogs, becomes increasingly important in achieving glycemic control. Metformin may still be used cautiously in patients with preserved renal function and low risk for lactic acidosis, while GLP-1 receptor agonists can be considered with close monitoring.

Other agents should be used selectively. DPP-4 inhibitors remain suitable with dose adjustments, and α-glucosidase inhibitors may help manage postprandial hyperglycemia. SGLT2 inhibitors can be used with caution due to the risk of volume depletion and acute kidney injury.

Sulfonylureas and glinides should be avoided because of their high risk of prolonged hypoglycemia, and pioglitazone is generally contraindicated owing to its potential to cause fluid retention and heart failure.

##### Patients with severe liver disease (Child-Pugh C)

In advanced liver disease, therapeutic options are significantly limited. Insulin therapy remains the safest and most effective option for glycemic control. Alpha-glucosidase inhibitors may be used cautiously to manage postprandial hyperglycemia.

All other oral antidiabetic agents are generally contraindicated because of unpredictable pharmacokinetics, heightened adverse event risk, and lack of safety data. Management should be reassessed frequently as hepatic function evolves, maintaining a low threshold for insulin initiation or dose adjustment as liver disease progresses.

#### Antidiabetic drug selection in specific clinical scenarios

Antidiabetic therapy in cirrhosis must be guided by individualized clinical assessment. Key factors, including renal impairment (eGFR), nutritional status (e.g., sarcopenia, malnutrition), and cirrhosis-related complications such as ascites, hepatic encephalopathy, HCC risk, and esophageal varices, play a critical role in determining drug choice, dosing, and safety.

Table 3 provides a summary of recommended antidiabetic agents across various clinical scenarios in cirrhotic patients. These recommendations are based on current evidence regarding pharmacokinetics, pharmacodynamics, and safety profiles within the context of liver dysfunction and its complications [66, 76, 95, 96].

**Table 3:** Antidiabetic drug selection in specific clinical scenarios

Clinical Scenario	Preferred Drug(s)	Used with Caution	Avoid	Notes
MASH/Obesity	Metformin, GLP-1 RAs, SGLT2 Inhibitors	Pioglitazone (due to weight gain and fluid retention)	Sulfonylureas (due to weight gain and hypoglycemia)	GLP-1 RAs and SGLT2 inhibitors offer weight loss and hepatic benefits. Metformin improves insulin sensitivity
Mild/Moderate CKD (eGFR > 30 mL/min/1.73 m²)	Insulin, DPP-4 inhibitors (linagliptin), GLP-1 RAs, Acarbose	Metformin (dose-adjust), other DPP-4 inhibitors (dose-adjust), SGLT2 inhibitors	Sulfonylureas	Linagliptin does not require renal adjustment. SGLT2 inhibitors have renal benefits but risk AKI. Metformin has lactic acidosis risk
Advanced CKD (eGFR < 30 mL/min/1.73 m²)	Insulin, Linagliptin, Acarbose	GLP-1 RAs (liraglutide and semaglutide, with careful monitoring)	Metformin, sulfonylureas, SGLT2 inhibitors, other DPP-4 inhibitors	Insulin is safest with renal failure. Acarbose is minimally absorbed. GLP-1 RAs require caution due to volume depletion risk
Sarcopenia / Malnutrition	Insulin, DPP-4 inhibitors, Acarbose	Metformin, GLP-1 RAs (can cause weight loss), SGLT2 inhibitors (volume depletion)	Sulfonylureas, Pioglitazone	Insulin promotes anabolism. Metformin and GLP-1 RAs/SGLT2 Inhibitors can worsen muscle loss. Pioglitazone causes fluid retention
Ascites / Edema	Insulin, Acarbose	DPP-4 inhibitors (decompensated cirrhosis), GLP-1 RAs (limited data), SGLT2 inhibitors (volume depletion)	Metformin, Sulfonylureas, Pioglitazone	Insulin does not exacerbate fluid retention. SGLT2 inhibitors may reduce ascites but increase AKI risk. Metformin and pioglitazone increase fluid retention
Hepatic Encephalopathy	Insulin, DPP-4 inhibitors, GLP-1 RAs, Acarbose	SGLT2 inhibitors (volume depletion)	Metformin (if overt HE), Sulfonylureas	Acarbose may reduce ammonia levels. Metformin is contraindicated in overt HE due to lactic acidosis risk. SGLT2 inhibitors may worsen HE via dehydration
HCC Risk	Metformin, GLP-1 RAs, SGLT2 Inhibitors	DPP-4 inhibitors, Pioglitazone, Acarbose	Sulfonylureas, Insulin (if possible)	Metformin, GLP-1 RAs, and SGLT2 inhibitors are associated with reduced HCC risk. Insulin and sulfonylureas may increase risk
Esophageal Varices	Insulin, DPP-4 inhibitors, GLP-1 RAs, Acarbose	Metformin (compensated), SGLT2 inhibitors (volume depletion)	Metformin (decompensated), Sulfonylureas, Pioglitazone	Insulin does not affect portal pressure. SGLT2 inhibitors may reduce portal pressure but risk AKI. Metformin increases lactic acidosis risk in decompensation

AKI: acute kidney injury; CKD: chronic kidney disease; DPP-4 inhibitors: dipeptidyl peptidase-4 inhibitors; eGFR: estimated glomerular filtration rate; GLP-1 RAs: glucagon-like peptide-1 receptor agonists; HCC: hepatocellular carcinoma; HE: hepatic encephalopathy; SGLT2 inhibitors: sodium-glucose cotransporter 2 inhibitors

#### Management during acute decompensation

Acute decompensation of cirrhosis presents unique challenges to diabetes management due to altered glucose metabolism, increased counter-regulatory stress hormones, and reduced hepatic glycogen stores [97]. During such episodes, insulin therapy becomes the mainstay, and all oral antidiabetic agents should be discontinued. Intravenous insulin infusion is the preferred method in critically ill patients due to frequent glycemic instability and the need for rapid, titratable control [98]. Close monitoring is essential, including blood glucose checks every 4–6 h and vigilance for hypoglycemia, especially in patients with poor intake. Nutritional support is critical to maintaining adequate caloric and protein intake, and insulin dosing should be adjusted accordingly [99].

#### Perioperative management

Perioperative diabetes management in cirrhotic patients requires thorough preoperative assessment of glycemic control, liver function, and nutritional status. Glycemic optimization is essential before elective procedures. All oral antidiabetic agents should be stopped 24–48 h before surgery. During surgery, blood glucose should be maintained between 140 and 180 mg/dL. Intravenous insulin infusion is preferred for major or prolonged surgeries, with continuous glucose monitoring intraoperatively [100, 101].

Postoperatively, frequent glucose monitoring should continue until oral intake stabilizes. Transition from IV to subcutaneous insulin should occur once the patient resumes eating [102].

#### Liver-specific procedures

Certain liver-directed interventions require specific attention in diabetic patients with cirrhosis. Transjugular intrahepatic portosystemic shunt (TIPS) may improve insulin sensitivity and reduce insulin requirements due to improved hepatic perfusion and reduced portal pressure [103]. In contrast, radiofrequency ablation (RFA) and transarterial chemoembolization (TACE) may lead to transient hyperglycemia as part of the acute stress response, necessitating temporary adjustments in insulin dosing [104, 105]. Large-volume paracentesis can increase the risk of hypoglycemia, especially in patients with limited oral intake or intensive insulin therapy, and thus requires close glucose monitoring before and after the procedure [106].

#### Post-liver transplantation management

Liver transplantation can dramatically alter glucose metabolism [107]. HD often improves or resolves post-transplant, with 50–70% of affected patients experiencing better glycemic control due to restored liver function [53]. However, PTDM occurs in 10–30% of recipients, primarily driven by immunosuppressive medications (especially corticosteroids and calcineurin inhibitors) [108].

In the early post-transplant period, frequent glucose monitoring is essential, and insulin remains the preferred initial therapy due to fluctuating metabolic needs. Oral agents may be introduced after stabilization, including GLP-1 receptor agonists such as semaglutide, which have demonstrated safety and efficacy in this population [109].

### Monitoring Strategies

Effective monitoring is essential for optimizing glycemic control while minimizing risks.

#### Self-Monitoring of Blood Glucose (SMBG)

In cirrhotic patients, SMBG is a cornerstone of diabetes care and generally requires more frequent checks than in non-cirrhotic individuals. Additional SMBG is recommended during periods of acute illness, medication adjustments, or changes in nutritional intake, as all of these can significantly alter glucose levels.

#### Continuous Glucose Monitoring (CGM)

CGM has become increasingly valuable in patients with cirrhosis due to their marked glycemic variability and higher incidence of asymptomatic or nocturnal hypoglycemia. Unlike SMBG, CGM provides real-time data and trend analysis, which enhances therapeutic precision and helps avoid both hypo- and hyperglycemia [110].

#### Hemoglobin A1c (HbA1c)

Hemoglobin A1c, while widely used to assess long-term glycemic control in the general population, is less reliable in cirrhosis. This inaccuracy stems from common hematologic abnormalities in liver disease, such as anemia, hypersplenism, frequent GI bleeding, and shortened red blood cell lifespan, all of which can lead to falsely low HbA1c readings. It is important to note that HbA1c may remain a clinically usable marker in selected patients with compensated cirrhosis who do not have anemia, hypersplenism, or other conditions affecting erythrocyte turnover. In contrast, exclusive reliance on HbA1c in advanced or decompensated cirrhosis may significantly underestimate true glycemic exposure, potentially delaying necessary treatment escalation [111].

#### Fructosamine and glycated albumin

Fructosamine and glycated albumin provide alternative measures of short-term glucose control over a 2–3 week period and are not influenced by red blood cell turnover. However, they are both affected by hypoalbuminemia and protein metabolism disturbances, which are frequent in cirrhosis [112]. Fructosamine reflects total glycated serum proteins and may be unreliable in patients with low albumin levels [113]. In contrast, glycated albumin offers a more specific assessment and has shown better correlation with CGM data, suggesting it may be a more dependable marker in this population.

## Current challenges and future perspectives in hepatogenous diabetes

### Knowledge gaps and areas of controversy

Despite growing recognition of HD as a distinct clinical entity, several important gaps and controversies remain. First, there is no universally accepted diagnostic criterion to clearly differentiate HD from T2DM in patients with cirrhosis. Optimal screening strategies are also unclear, including the most appropriate diagnostic test, screening intervals, and target populations.

Glycemic targets in HD are not well defined, as current evidence is insufficient to guide the balance between effective glucose control and the risk of hypoglycemia. Moreover, HD is not yet formally recognized by major diabetes organizations, which limits awareness and hinders the development of dedicated clinical guidelines.

### Future directions

Advances in the management of HD will increasingly rely on precision medicine, enabling individualized therapies tailored to genetic backgrounds, liver disease etiologies, and specific metabolic phenotypes. Central to this progress is the identification of novel, non-invasive biomarkers, such as specific hepatokines or gut microbiota signatures, that can reliably distinguish HD from T2DM and predict its development. Furthermore, developing and validating simplified, risk-stratified screening tools for use in hepatology clinics is essential to improve clinical recognition. Establishing evidence-based clinical practice guidelines specifically for HD, stratified by Child-Pugh class and etiology, will allow the field to move beyond simply adapting T2DM protocols to a more specialized standard of care.

Future therapeutic strategies must emphasize early prevention and the integration of multidisciplinary care to optimize patient outcomes. Combination therapies targeting multiple pathogenic pathways, alongside innovations such as smart insulin delivery systems, hold the potential to optimize glycemic control while minimizing adverse effects like hypoglycemia. Additionally, addressing sarcopenia through myostatin inhibition, leucine-enriched amino acid supplementation, and structured exercise programs offers a promising avenue for improving insulin sensitivity and broader metabolic outcomes [114]. To support these clinical advances, large-scale randomized controlled trials of newer agents, such as GLP-1 receptor agonists and SGLT2 inhibitors, are urgently needed in patients with Child-Pugh B and C cirrhosis to establish definitive safety and efficacy in advanced disease.

## Conclusion

Hepatogenous diabetes is a significant, yet often underrecognized, complication of liver cirrhosis. It presents unique challenges in diagnosis and management due to its distinct pathophysiological features and overlap with other forms of diabetes. Its severe insulin resistance, high risk of hypoglycemia, and unique complications profile distinguish it from T2DM. A shift toward precision-based, multidisciplinary care, along with continued research into targeted therapies and preventive strategies, is essential to improve outcomes in this growing patient population.

## Data Availability

No datasets were generated or analysed during the current study.

## Abbreviations

β-cell: Beta Cell
CLD: Chronic Liver Disease
DM: Diabetes Mellitus
DPP-4: Dipeptidyl Peptidase-4
FGF21: Fibroblast Growth Factor 21
GLP-1: Glucagon-Like Peptide-1
GLUT4: Glucose Transporter Type 4
HbA1c: Glycated Hemoglobin
HD: Hepatogenous Diabetes
HOMA-IR: Homeostatic Model Assessment of Insulin Resistance
IGF-1: Insulin-Like Growth Factor 1
IGT: Impaired Glucose Tolerance
IR: Insulin Resistance
MASLD: Metabolic Dysfunction–Associated Steatotic Liver Disease
MASH: Metabolic Dysfunction–Associated Steatohepatitis
NGT: Normal Glucose Tolerance
OGTT: Oral Glucose Tolerance Test
PTDM: Post-Transplant Diabetes Mellitus
SGLT2: Sodium–Glucose Transport Protein 2
T2DM: Type 2 Diabetes Mellitus

## Declarations

### Generative AI and AI-assisted technologies in the manuscript preparation process

During the preparation of this work, the authors used Perplexity in order to assist with language editing support. After using this tool, the authors reviewed and edited the content as needed and take full responsibility for the content of the published article.
